# The "silver" Japanese quail and the *MITF *gene: causal mutation, associated traits and homology with the "blue" chicken plumage

**DOI:** 10.1186/1471-2156-11-15

**Published:** 2010-02-25

**Authors:** Francis Minvielle, Bertrand Bed'hom, Jean-Luc Coville, Shin'ichi Ito, Miho Inoue-Murayama, David Gourichon

**Affiliations:** 1 UMR 1313 INRA/AgroParisTech, Génétique animale et biologie intégrative GABI, F-78352, Jouy-en-Josas, France; 2 Faculty of Applied Biological Sciences, Gifu University, Gifu 501-1193, Japan; 3 Wildlife Research Center of Kyoto University, Kyoto 606-8203, Japan; 4 UE 1295 INRA, PEAT, 37380 Nouzilly, France

## Abstract

**Background:**

The *MITF *(*microphthalmia-associated transcription factor*) gene has been investigated in mice and various vertebrates but its variations and associated effects have not yet been explored much in birds. The present study describes the causal mutation *B *at the *MITF *gene responsible for the "silver" plumage colour in the Japanese quail (*Coturnix japonica*), and its associated effects on growth and body composition, and tests its allelism with the "blue" plumage colour mutation *Bl *in *Gallus gallus*.

**Results:**

The semi dominant *B *mutation results from a premature stop codon caused by a 2 bp deletion in exon 11 of *MITF*. Homozygous "white" (*B/B*) quail which have a white plumage also show a slightly lower growth, lower body temperature, smaller heart, and lighter *pectoralis *muscles but more abdominal adipose tissue than the recessive homozygous "wild-type" (*+/+*) and heterozygous "silver" (*B/+*) quail. Similar observations on cardiac and body growth were made on mice (*Mus musculus*) homozygous for mutations at *MITF*. The production of chicken-quail hybrids with a white plumage obtained by crossing *Bl/+ *chicken heterozygous for the *blue *mutation with *B/B *white quail indicated that the mutations were allelic.

**Conclusion:**

The "silver" Japanese quail is an interesting model for the comparative study of the effects of *MITF *in birds and mammals. Further investigation using a chicken family segregating for the "blue" plumage and molecular data will be needed to confirm if the "blue" plumage in chicken results from a mutation in *MITF*.

## Background

**Figure 1:**
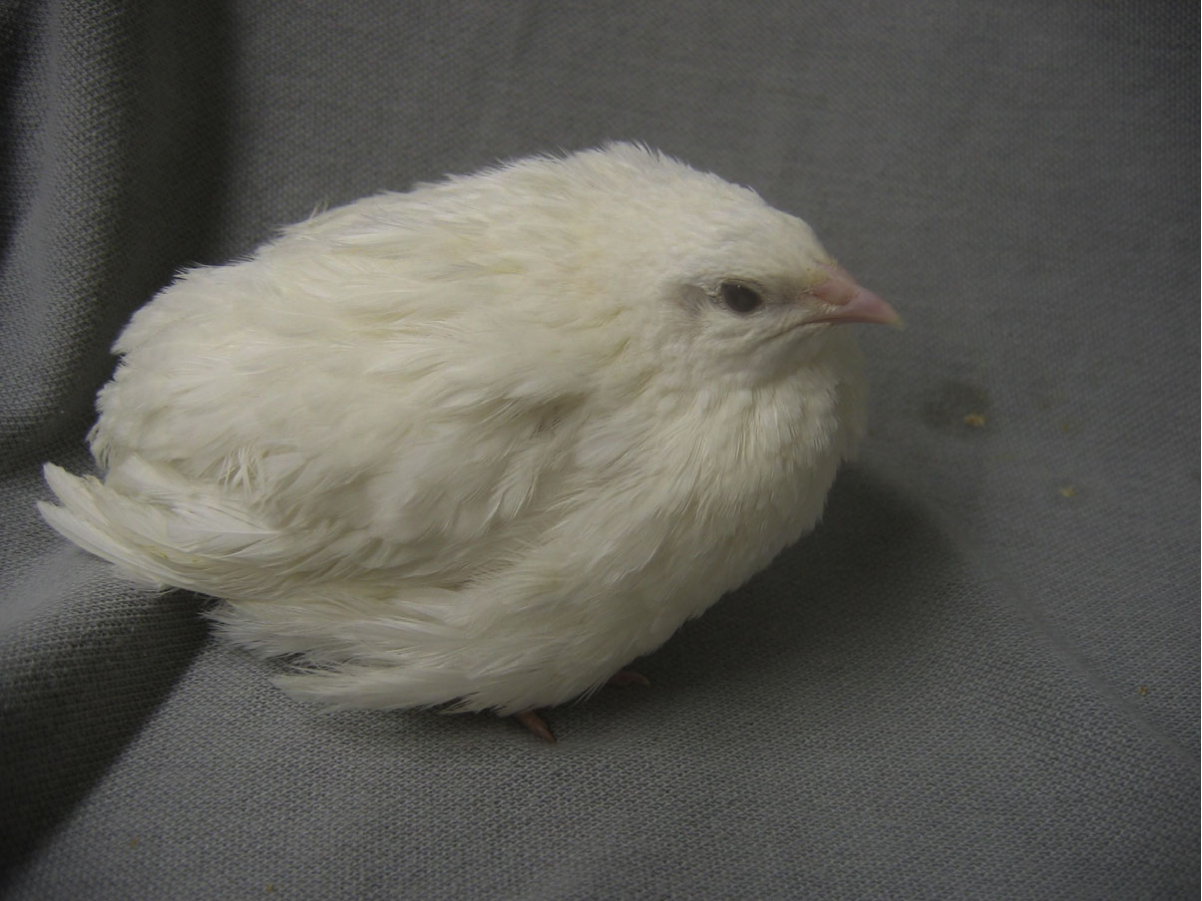
**Japanese quail homozygous for the "*silver" *mutation *B***. "White" quail have a snow-like white plumage colour and are homozygous (*B/B*) for the semi dominant *silver *mutation *B *localised in the *MITF *gene.

**Figure 2:**
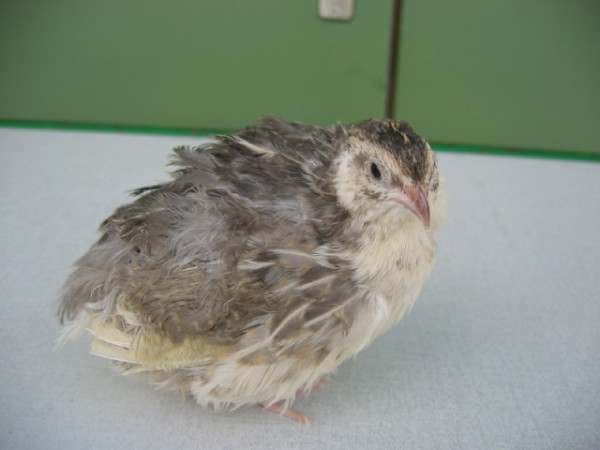
**Japanese quail heterozygous for the "*silver" *mutation *B***. "Silver" quail have a diluted grey plumage colour and are heterozygous (*B/+*) for the semi dominant *silver *mutation *B *localised in the *MITF *gene.

MITF (microphthalmia-associated transcription factor) is a member of the bHLH-leucine zipper transcription factor family and is involved in the development of melanocytes, retinal cells, osteoclasts and mast cells [1]. Mutations at the *MITF *gene have been described in seven vertebrate species [1,2], including *Coturnix japonica *[3]. Studies on mice have shown the existence of many alleles at this locus, and semi dominant mutations like *Mitf*^*Mi*-*wh *^produce heterozygous mice with a diluted grey coat colour and homozygous mice which are completely white [4]. These mutations have detrimental effects on melanocytes and lead to decreased pigmentation and various defects which have been extensively reported in mice but little investigated in other animal species [5]. Similarly, the "white" Japanese quail [6] homozygous for the semi dominant "*silver" *plumage colour mutation (*B*) have a white plumage colour (Figure 1) and heterozygotes (*B/+*) have a diluted grey "silver" plumage (Figure 2). The plumage colour of the "white" and "silver" Japanese quail was found previously to be associated with changes in the sequence of *MITF *in two different regions of the coding sequence, but the change that affects *MITF *activity has not yet been determined [3], and, apart from osteopetrosis [7], other phenotypic effects associated with the mutation have not yet been studied in *Coturnix*. In *Gallus*, *MITF *has been sequenced [8] but no associated plumage colour variation has been reported so far. Yet, the *blue *mutation (*Bl*) first reported in the rare Andalusian chicken breed [9] would be a likely candidate for *MITF *induced variation in *Gallus *because this semi dominant mutation is the only one reported in the chicken that produces white homozygous and greyish blue heterozygous birds (Additional file 1: Male chicken heterozygous and homozygous for the *blue *mutation *Bl*).

The present work on *Coturnix japonica *had three objectives. First, we intended to locate the region in the coding sequence which is responsible for the "silver" plumage colour (*B/+*) in the Japanese quail. Second, we studied the phenotypic consequences of the mutation by comparing the three genotypes (*B/B*, *B/+*, *+/+*) for several quantitative traits related to growth, food intake, metabolism and body composition. Finally, we tested the allelism between the *silver *(*B*) and *blue *(*Bl*) mutations by producing chicken-quail hybrids between homozygous *B/B *white female quail and heterozygous *Bl/+ *cocks.

## Results and Discussion

### Localization of the quail "silver" mutation in MITF

The structure of the quail *MITF *gene in 11 exons is described in Additional file 2. By alignment with chicken genomic sequence, only 10 exons could be identified in the single quail mRNA sequence AB005229, but since the information provided by the chicken coding sequences was compatible with the existence of 11 exons, they were numbered according to this organisation. Moreover, in other vertebrate species *MITF *has several transcript isoforms [1], and the only mRNA sequence published for quail is probably not sufficient to characterise all possible exons for *MITF*. This annotation suggested that the non-synonymous change and the deletion described previously in "silver" quail [3] were respectively in exons 8 and 11 of *MITF*. To assess the presence of these putative causal mutations in our quail population, we sequenced these *MITF *regions from genomic DNA of 3 recessive homozygous *+/+ *and 3 homozygous *B/B *quails. The sequences obtained for the quail were deposited in the GenBank nucleotide database under accession numbers GQ386796-GQ386799.

We were able to confirm that the *silver *allele *B *is associated with the 2 bp deletion in exon 11, because the 3 "wild-type" (*+/+*) quail were homozygous for the undeleted sequence, and the 3 "white" (*B/B*) birds were homozygous for the 2 bp deletion, but we did not find the association with the non-synonymous change in exon 8 reported previously [3]. The absence of polymorphism at this position in our population segregating for *silver *may be the consequence of a previous recombination event. Next, the 2 bp deletion polymorphism was genotyped in 86 "white", "silver" and "wild-type" quail. The genotypes were fully in agreement with the observed phenotypes: "wild-type" quail were homozygous for the undeleted sequence, "white" birds were homozygous for the deleted sequence and animals with the grey "silver" plumage had both alleles. This perfect association confirmed that the premature stop codon resulting from the 2 bp deletion in exon 11 was the most probable cause of the *B *mutation.

### Phenotypic effects associated with the "silver" mutation

Parameters of the monomolecular growth curves [10] estimated for the three plumage colour genotypes (*B/B*, *B/+*, *+/+*) are given and compared in Table 1. The coefficients of determination were high and similar for the three genotypes and indicated that the fit was satisfactory. The range of body weight *Bw *and the relative rate of growth *k *were smaller for the "white" quail (p < 0.05 and p < 0.01, respectively) indicating that the *B/B *genotype was associated to lower growth until maturity. The measures taken during the 3-week feed trial carried out on adult quails are listed in Table 2. The differences in body weight were confirmed, and *B/B *quail had both lower feed intake (p < 0.01) and body weight (p < 0.01) than *+/+ *quail, whereas the *B/+ *quail were not different from the *+/+ *ones. Egg number and weight during the test were not affected by the genotype at *MITF*. Rectal temperature of fasted or unfasted "white" quail was lower (p < 0.001 and p < 0.01, respectively) than that of wild-type quail, and, but to a lesser extent, of "silver" heterozygous birds. The results of the gross dissection of quail carcasses are listed and compared on an equal carcass weight basis in Table 3. "White" quail had more abdominal adipose tissue (p < 0.01) but less *pectoralis *muscles (p < 0.001) and smaller heart (p < 0.01) than the other two genotypes which were similar for these three traits. Liver weight was similar for all three genotypes. Tibia weight was higher in *B/B *quail than in the other two genotypes (p < 0.001). Our results on growth were consistent with the decreased body size reported for other quail plumage colour mutations like *roux *[11] and for dominant homozygous white mice [12], but our data on decreased feed intake and body temperature did not seem to have any equivalent in the Literature on *MITF *in mice. It does not seem that body composition has been studied in *MITF *mutant mice except for heart and body weight [13]. The results on *B/B *quail heart size were very much like those obtained in homozygous *MITF*-mutated mice [13] which have lower heart weight, and indicate that *MITF *is also associated to cardiac growth in birds. Our data on *pectoralis *muscles also show that *MITF *might be involved in skeletal muscle growth, since *B/B *quail had significantly lighter *pectoralis *muscles, but published reports on expression of *MITF *in mice skeletal muscles could not be found for comparison purposes. Our results on the heavier tibia weight of "white" quail were in agreement with the increased calcification in the tibias of quail [7] and mice [14] homozygous for the strong semi dominant *MITF *mutations, reported previously.

**Table 1:** Parameters (mean ± SD) and coefficients of determination of the growth curves^1 ^for "white" homozygous (*B/B*), "silver" heterozygous (*B/+*) and "wild-type" recessive homozygous (*+/+*) Japanese quail, effects of the family, sex, and genotype on the parameters of individual curves, and comparisons between plumage colour genotypes

Trait	Genotype						Anova	Significance of main effects			Contrast		
	
	White (*B*/*B*)		Silver (*B*/+)		Wild-type (+/+)		*R*^2^	Family	Sex	Genotype	(*B*/*B*)-(+/+)	(*B*/+) -(+/+)	(*B*/*B*)-(B/+)
*A*	179.7 ± 29.2		179.8 ± 19.9		183.5 ± 18.9		0.79	*	***	NS	NS	NS	NS

*Bw*	191.8 ± 31.4		196.1 ± 21.5		199.3 ± 19.9		0.80	**	***	*	-12.8*	NS	-11.5*

*k*	0.0376 ± 0.0093		0.0454 ± 0.0063		0.0447 ± 0.0050		0.62	NS	***	**	-0.0067**	NS	-0.0068**

CD^2^	0.85		0.92		0.93		--	--	--	--	--	--	--

^1^: Obtained with the monomolecular model: body weight = *A*-*Bw *exp(-*kt*), where *A *is the asymptotic body weight (g), *Bw *is the range of body weight from hatching to asymptotic weight, *k *is the relative rate of growth, and *t *is the age in days.

^2^: CD = coefficient of determination.

*:p < 0.05; **:p < 0.01; ***:p < 0.001; NS: not significant.

**Table 2:** Effects of family, sex, and plumage colour genotype (*B/B*, *B/+*, and *+/+*) on body weights, feed intake, egg production and body temperature (mean ± SD) of 9-week old Japanese quail, and comparisons between genotypes

Trait	Genotype						Anova	Significance of main effects			Contrast		
	
	White (*B*/*B*)		Silver (*B*/+)		Wild-type (+/+)		*R*^2^	Family	Sex	Genotype	(*B*/*B*)-(+/+)	(*B*/+) -(+/+)	(*B*/*B*)-(B/+)
64-d BW after 12 h fasting (g)	148.5 ± 20.2		154.5 ± 15.6		158.2 ± 15.1		0.71	*	***	**	-14.9***	NS	-11.9**

85-d BW after 12 h fasting (g)	159.8 ± 23.5		161.4 ± 16.6		164.5 ± 16.2		0.73	**	***	*	-11.3**	NS	-8.4*

Daily feed intake (g)	20.5 ± 5.2		21.1 ± 4.0		22.4 ± 5.1		0.78	*	***	**	-3.43**	NS	-2.45*

BW gain on 3-wk feed test (g)	11.3 ± 7.4		6.9 ± 6.6		6.3 ± 5.8		0.26	NS	NS	NS	NS	NS	NS

Egg number on 3-wk test	17.0 ± 6.1		19.1 ± 2.9		18.9 ± 4.3		0.37	NS	-	NS	NS	NS	NS

Egg weight (g)	9.87 ± 0.87		9.61 ± 0.71		9.73 ± 0.52		0.59	*	-	NS	NS	NS	NS

64-d body temperature (°C)	40.82 ± 0.40		41.16 ± 0.32		41.32 ± 0.29		0.50	NS	NS	***	-0.53***	-0.20**	-0.33**

85-d body temperature (°C)	40.67 ± 0.25		41.23 ± 0.35		41.34 ± 0.33		0.51	NS	NS	***	-0.57***	NS	-0.44***

90-d body temperature (°C)	41.19 ± 0.36		41.62 ± 0.35		41.74 ± 0.36		0.39	NS	*	*	-0.44**	NS	-0.34*

*:p < 0.05; **:p < 0.01; ***:p < 0.001; NS: not significant.

**Table 3:** Effects of family, sex and plumage colour genotype (*B/B*, *B/+*, and *+/+*) on gross body composition (mean ± SD) of 24-week old Japanese quail, and comparisons between genotypes

Trait	Genotype						Anova	Significance of main effects			Contrast		
	
	White (*B*/*B*)		Silver (*B*/+)		Wild-type (+/+)		*R*^2^	Family	Sex	Genotype	(*B*/*B*)-(+/+)	(*B*/+)-(+/+)	(*B*/*B*)-(*B*/+)
Carcass weight (g)	159.8 ± 27.9		163.2 ± 19.4		168.3 ± 18.7		0.80	**	***	**	-16.6**	-6.7*	NS

Abdominal adipose tissue^1 ^(g)	2.3 ± 3.2		1.4 ± 0.9		1.5 ± 1.3		0.69	NS	***	*	1.5**	NS	1.2**

*Pectoralis *muscle weight^1,2 ^(g)	11.7 ± 2.2		13.0 ± 1.5		13.4 ± 1.6		0.79	NS	NS	**	-1.9***	NS	-1.6***

Liver weight^1 ^(g)	3.4 ± 1.1		3.6 ± 1.4		3.5 ± 1.0		0.77	NS	***	NS	NS	NS	NS

Heart weight^1 ^(g)	1.2 ± 0.2		1.4 ± 0.2		1.5 ± 0.2		0.61	NS	NS	*	-0.2**	NS	-0.2**

Tibia weight (g)	0.91 ± 0.19		0.72 ± 0.10		0.70 ± 0.08		0.66	***	***	***	0.17***	NS	0.15***

^1^:Carcass weight was used as a covariable for the analyses of abdominal adipose tissue, *pectoralis *muscle weight, liver weight, and heart weight.

^2^:Total weight of the right *pectoralis major *and *pectoralis minor *muscles.

*:p < 0.05; **:p < 0.01; ***:p < 0.001; NS: not significant.

### Homology with the "blue" Gallus plumage colour mutation

**Figure 3:**
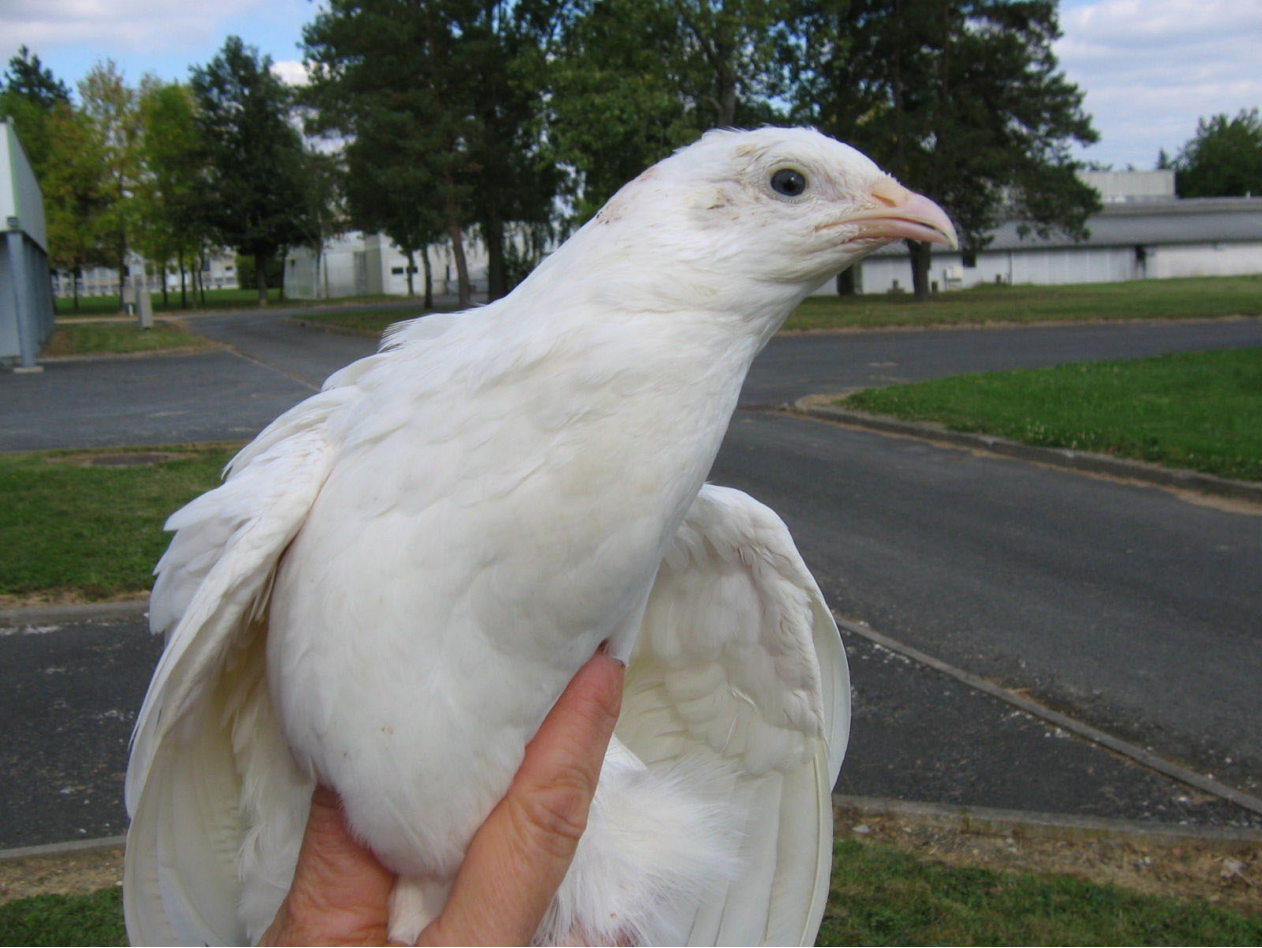
**Chicken-quail hybrid with a "white" plumage**. This is the adult progeny of the cross between a "white" *B/B *female *Coturnix *and a *Bl/+ *male *Gallus *with a "blue" plumage. It shows a white "silver"quail-like plumage that would be expected for hybrids with the *Bl/B *genotype if *blue *and *silver *were allelic mutations.

**Figure 4:**
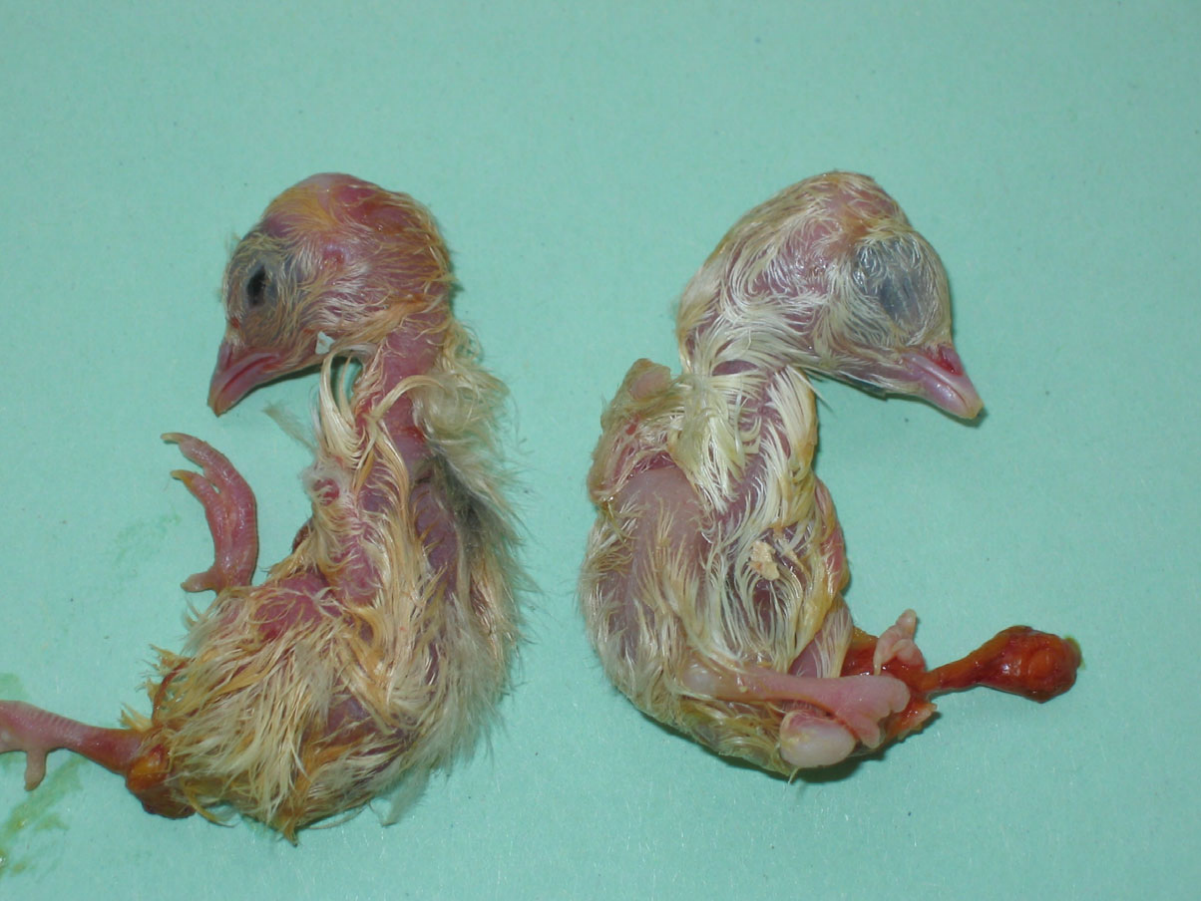
**Pigmented and "white" hybrid embryos**. They are the unhatched progeny of the cross between a *B/B *female *Coturnix *and a *Bl/+ *male *Gallus *with a "blue" plumage. The embryo on the left shows greyish feathers, and the embryo on the right has apparently only whitish feathers, the only two plumage colours that would be expected in *+/B *and *Bl/B *hybrids if *blue *and *silver *were allelic mutations.

By crossing blue heterozygous (*Bl/+*) chicken males with *B/B *quail females, variously coloured hybrids, but no uniformly white ones, were expected if the gene for the *blue *mutation was not *MITF*. On the contrary, if it was, only two hybrid genotypes at *MITF *were expected: *Bl/B *and *+/B*, with respectively a white and a "silver" plumage. Out of the 582 eggs which were incubated, two non-sib hybrids with a uniformly white plumage hatched alive. One died accidentally 4 days later but the other one survived for over a year (Figure 3). Fifteen other eggs had also been fertilised, but did not hatch. Their contents were checked visually, and both pigmented and white feathered embryos were observed (Figure 4). These observations are a strong indication that the "blue" plumage colour in *Gallus *is due to a mutation in the *MITF *gene.

## Conclusion

The 2 bp deletion in exon 11 of *MITF *produces the *silver B *mutation, with phenotypic consequences on cardiac growth and bone development which are very similar to those described in mice homozygous for semi dominant *MITF *mutations. Our results on decreased skeletal muscle growth and lower body temperature are quite original, however, and these traits should also be investigated in *MITF*-mutated mutant mice to contribute to the comparative study of the effects of this gene in birds and mammals. Except for one measure of body temperature, *B/+ *and +/+ quail had similar performances which indicate that the "*silver" *mutation could be recessive for most traits but plumage colour. Further investigations on "white" and "blue" plumage colour variation in *Gallus *are needed to study the association between markers in *MITF *and plumage colour in chickens with the *blue *mutation.

## Methods

### Birds

The *silver *mutation was introduced from Gifu University (Japan) in 2003, and 4 *B/+ *females from two different full sib families were obtained. They were crossed to 4 males from a local wild-type plumage experimental line to found a quail line segregating for *B*, but *+/+ *for other known quail plumage colour genes, which was set up in the INRA PEAT experimental unit in Nouzilly, France. The present study was carried out 10 generations after the initial importation, and the experimental population used in this work was the progeny (n = 90) of 17 single pair matings between *B/+ *quail. All quail were produced in a single hatch and raised together with free access to *ad libitum *commercial feed and drinking water, in accordance with French regulations.

Chicken-quail hybrids were produced in PEAT by artificial insemination of *B/B *homozygous quail with mixed sperm from three "blue" heterozygous *Bl/+ *cocks that had been identified by their greyish blue feathering.

### Traits

All quail were weighed weekly until 6 weeks of age. From 9 to 12 weeks of age they underwent a feed trial during which their individual feed intake was monitored, rectal body temperature and body weight were measured twice, and, in females, egg production and weight were registered. At the age of 24 weeks, they were sacrificed using authorised procedures, and a gross dissection was performed. The weights of each carcass, abdominal adipose tissue, liver, heart, and right *pectoralis major *and *pectoralis minor *muscles were measured, and 50 right tibias from quail belonging to 15 families were collected, cleaned, and weighed.

### Statistical analyses

Quail growth was studied using the nonlinear monomolecular model [10]: body weight = *A *- *Bw *exp(-*kt*), where *A *is the asymptotic body weight, *Bw *is the range of body weights from hatching to asymptotic body weight, *k *is the relative rate of growth, and *t *is the age in days, and the adjustment was carried out with the NLIN procedure [15]. Parameters of individual growth curves, body weights, feed intake and carcass weight were analysed by analysis of variance (ANOVA) with family, sex and genotype *(B/B*, *B/+*, or *+/+*) as the three main effects and without interactions, because first-order interactions were found not to be significant in preliminary analyses. The linear model used for the ANOVA of egg number and egg weight was similar but did not include the effect of sex. Body temperatures and dissection traits were analysed by an analysis of covariance (ANCOVA), with the three same main effects as for the ANOVA plus contemporaneous body weight (for measures of rectal temperature) or carcass weight (for dissection traits) as a covariable. Tibia weight was analysed by both ANOVA and ANCOVA. Contrasts between least-squares means for the three genotypes at *MITF *were estimated from the analyses and tested. Significance was set at p < 0.05. These analyses were carried out with the GLM procedure [15].

### Sequencing and genotyping

Blood samples have been taken from all the quail. Crude DNA extraction was conducted with 2 μL of whole blood incubated with 250 μL of NaOH (0.2 M) at 65°C for 2 h, followed by neutralization with 250 μL of Tris-HCl (0.2 M). The structure of the *MITF *gene in quail was determined by comparative alignment with chicken genomic and coding sequences (mRNA and ESTs) for *MITF*, using the quail mRNA sequence (AB005229). The coding sequences were aligned on the second assembly of the chicken genome using the EST2PCR software from the EMBOSS package [16]. The comparison with the chicken genome suggests that the non-synonymous change occurred in exon 8, and that the deletion occurred in exon 11, the last exon of *MITF*.

For a region encompassing the end of exon 8, all intron 8 and beginning of exon 9, DNA was amplified using primers MITF_SF (5'TCCTACAGAGTCAGAAGCGAGA-3') and MITF_SR (5'-GGTATCAAGGTGCCCAGTTC-3'), leading to a product of 1055 bp which was sequenced using PCR primers and an additional internal primer, MITF_SR2 (5'-CAGCAGCACCTTTGAGAACA-3'). For the region of exon 11, DNA was amplified using primers MITF2_SF (5-AGCTCGGGCACATGGACT-3') and MITF2_SR (5'-GGAGAGGGTATCGTCCATCA-3') leading to a product of 278 or 280 bp according to the genotype, and sequenced using PCR primers. Next, the 2 bp deletion polymorphism in exon 11 was genotyped in all quails by capillary electrophoresis using an automated ABI3730 sequencer, after PCR with primers amplifying a region encompassing this deletion: MITF_Genot_F (5'-CTGTCCCTTGTTCCATCCAC-3') and MITF_Genot_R_FAM (5'-FAM-TTGGTTGCAGTTATCCAGCA-3'). The observed size of PCR products differed between genotypes. It was 90 bp for *+/+*, 88 bp for *B/B*, and 88 and 90 bp for *B/+ *quail.

## Authors' contributions

FM coordinated the study and wrote the paper, BB coordinated the sequencing and the genotyping and contributed to the redaction of the paper, J-LC carried out the sequencing and the genotyping, SI and MI-M contributed the silver quail stock and participated in the redaction of the paper, and DG supervised the production and the phenotyping of quail and hybrids and participated in the redaction of the paper. All authors read and approved the final manuscript.

## Supplementary Material

Additional file 1 **Male chicken heterozygous and homozygous for the *blue *mutation *Bl***. The heterozygous *Bl/+ *male (on the left) has a "blue" plumage, and the homozygous *Bl/Bl *(on the right) has a white plumage with some pigmented feathers. Click here for file

Additional file 2 **The structure in 11 exons of the *MITF *gene in the Japanese quail (*Coturnix japonica*)**. Click here for file
