# Supplementary figures and images for: The "silver" Japanese quail and the MITF gene: causal mutation, associated traits and homology with the "blue" chicken plumage
Source: BMC Genet. 2010 Feb 25;11:15. doi: 10.1186/1471-2156-11-15 (PMC2841575; doi:10.1186/1471-2156-11-15)

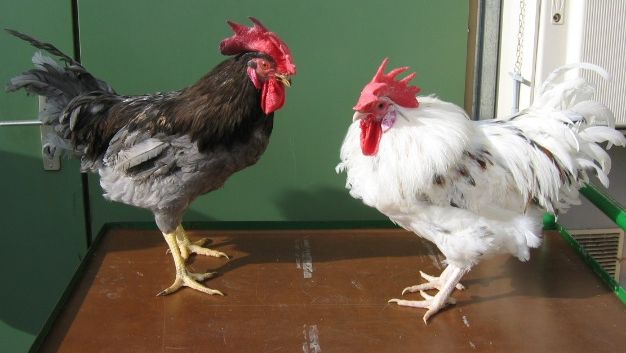

Supplement: Additional file 1 — Male chicken heterozygous and homozygous for the blue mutation Bl. The heterozygous Bl/+ male (on the left) has a "blue" plumage, and the homozygous Bl/Bl (on the right) has a white plumage with some pigmented feathers. [file 1471-2156-11-15-S1.JPEG]
