# Supplementary material for: The "silver" Japanese quail and the MITF gene: causal mutation, associated traits and homology with the "blue" chicken plumage
Source: BMC Genet. 2010 Feb 25;11:15. doi: 10.1186/1471-2156-11-15 (PMC2841575; doi:10.1186/1471-2156-11-15)
Supplement: Additional file 2 — The structure in 11 exons of the MITF gene in the Japanese quail (Coturnix japonica). [file 1471-2156-11-15-S2.DOC]

The structure of the *MITF* gene has been determined by alignment to the chicken genome of *MITF* coding sequences from chicken and quail available in the GenBank database, using the EST2GENOME software from the EMBOSS package. Putative exons are represented in light blue on the region chr12:15,598,125-16,049,835) from the May 2006 Chicken assembly. Quail coding sequences are represented in red, and Chicken coding sequences are represented in green.
